# HIV-2/SIV Vpx antagonises NF-κB activation by targeting p65

**DOI:** 10.1186/s12977-021-00586-w

**Published:** 2022-01-24

**Authors:** Douglas L. Fink, James Cai, Matthew V. X. Whelan, Christopher Monit, Carlos Maluquer de Motes, Greg J. Towers, Rebecca P. Sumner

**Affiliations:** 1 Division of Infection and Immunity, University College London, 90 Gower Street, London, WC1E 6BT UK; 2 Department of Microbial Sciences, School of Biosciences and Medicine, University of Surrey, Guildford, UK

**Keywords:** Vpx, NF-κB, p65, HIV-2, SIV, Immunomodulator

## Abstract

**Background:**

The NF-κB family of transcription factors and associated signalling pathways are abundant and ubiquitous in human immune responses. Activation of NF-κB transcription factors by viral pathogen-associated molecular patterns, such as viral RNA and DNA, is fundamental to anti-viral innate immune defences and pro-inflammatory cytokine production that steers adaptive immune responses. Diverse non-viral stimuli, such as lipopolysaccharide and cytokines, also activate NF-κB and the same anti-pathogen gene networks. Viruses adapted to human cells often encode multiple proteins targeting the NF-κB pathway to mitigate the anti-viral effects of NF-κB-dependent host immunity.

**Results:**

In this study we have demonstrated using a variety of assays, in a number of different cell types including primary cells, that plasmid-encoded or virus-delivered simian immunodeficiency virus (SIV) accessory protein Vpx is a broad antagonist of NF-κB signalling active against diverse innate NF-κB agonists. Using targeted Vpx mutagenesis, we showed that this novel Vpx phenotype is independent of known Vpx cofactor DCAF1 and other cellular binding partners, including SAMHD1, STING and the HUSH complex. We found that Vpx co-immunoprecipitated with canonical NF-κB transcription factor p65, but not NF-κB family members p50 or p100, preventing nuclear translocation of p65. We found that broad antagonism of NF-κB activation by Vpx was conserved across distantly related lentiviruses as well as for Vpr from SIV Mona monkey (SIVmon), which has Vpx-like SAMHD1-degradation activity.

**Conclusions:**

We have discovered a novel mechanism by which lentiviruses antagonise NF-κB activation by targeting p65. These findings extend our knowledge of how lentiviruses manipulate universal regulators of immunity to avoid the anti-viral sequelae of pro-inflammatory gene expression stimulated by both viral and extra-viral agonists. Importantly our findings are also relevant to the gene therapy field where virus-like particle associated Vpx is routinely used to enhance vector transduction through antagonism of SAMHD1, and perhaps also through manipulation of NF-κB.

**Graphical Abstract:**

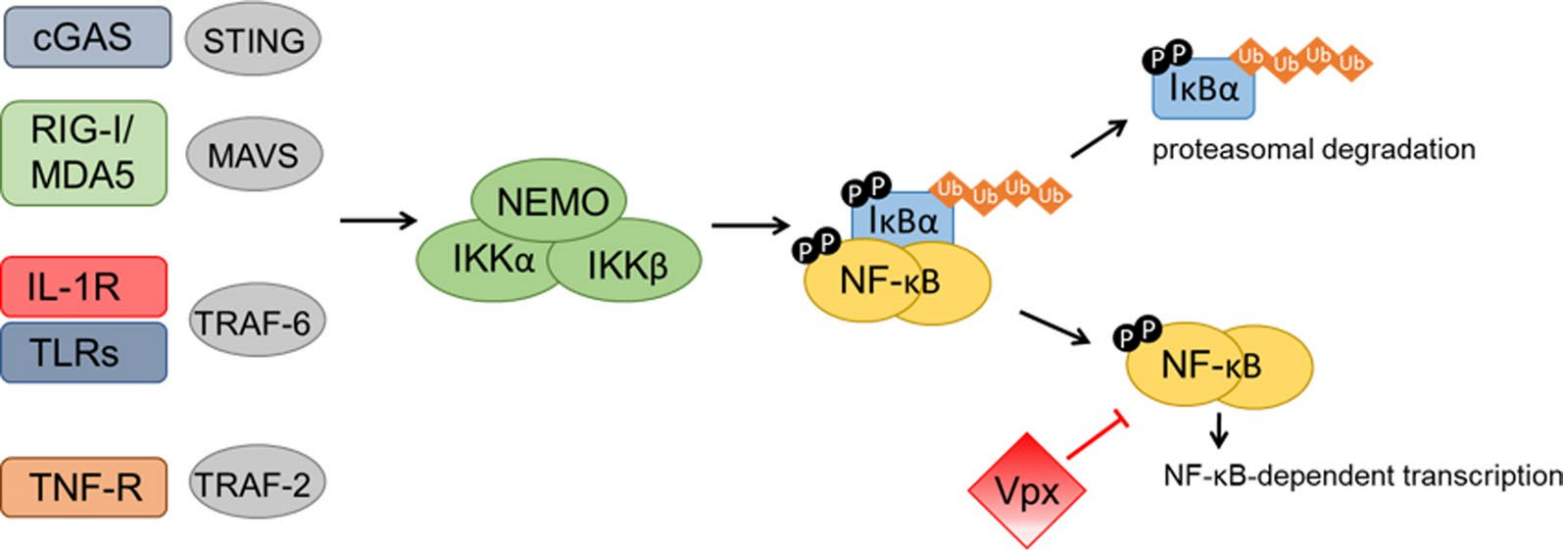

**Supplementary Information:**

The online version contains supplementary material available at 10.1186/s12977-021-00586-w.

## Background

Nuclear factor kappa B (NF-κB) transcription factors (TFs) are pivotal mediators of inflammation [1]. Amongst diverse roles in immune signalling, NF-κB activation is fundamental to pro-inflammatory anti-viral innate defences. Viruses typically activate NF-κB through detection of viral pathogen-associated molecular patterns (PAMPs) by host pattern-recognition receptors (PRRs), such as Toll-like receptors, retinoic acid-inducible gene (RIG)-like receptors and DNA sensors (such as the cyclic GMP-AMP synthase (cGAS) and stimulator of interferon genes (STING) pathway) [2–5]. Canonical NF-κB activation by PRRs, and other diverse ligands such as cytokines, leads to degradation of inhibitory proteins (such as IκB) and release of NF-κB TF dimers, predominantly p50 and p65 (RelA), which translocate into the nucleus to upregulate an array of genes that govern cell-autonomous and adaptive responses against infection [1, 6, 7]. Non-canonical NF-κB pathways are activated by a more narrow range of stimuli, including a subset of the TNF receptor superfamily members such as CD40, and depend on processing of TF p52 precursor protein, p100 [8].

Emphasising the primacy of NF-κB TFs to host defences, viruses have evolved strategies to antagonise NF-κB signalling, often targeting pathway activation at multiple levels in complex ways [2]. HIV-1 deploys at least two direct NF-κB antagonists which target multiple points of NF-κB signalling. Accessory protein Vpu, encoded by HIV-1 and its ancestor lentivirus, chimpanzee simian immunodeficiency virus (SIVcpz), antagonises NF-κB through at least two mechanisms. Vpu directly binds and inhibits tetherin, a host restriction factor that inhibits lentiviral budding and also activates NF-κB on virus engagement [9–11]. Independently of tetherin, Vpu also stabilises IκB to inhibit p65 nuclear translocation and NF-κB-activated transcription [12–15]. In contrast, HIV-2 does not possess a *vpu* gene and tetherin antagonism is effected through HIV-2 Env which intriguingly activates rather than inhibits NF-κB signaling [16]. HIV-1 also suppresses NF-κB activation at the level of TF nuclear transport, through accessory protein HIV-1 Vpr interaction with karyopherins [17, 18]. This equivalent activity of HIV-1 Vpu or Vpr as broad NF-κB antagonists has not been studied for HIV-2.

HIV-2-related SIVsm/SIVmac and SIVrcm/SIVmnd-2 lineage lentiviruses, which infect sooty mangabey, macaque, red-capped mangabey and mandrill monkeys respectively, all of which belong to the same primate family, possess two homologous genes to HIV-1/SIVcpz *vpr*: *vpr* and *vpx* [19, 20]. Typically if a virus has 2 Vpr-like genes, one of them is named Vpx. That is, no viruses have been assigned a Vpx without a Vpr. But the identification of a particular gene as a Vpr or a Vpx is complex because high levels of adaptation prevent alignment and effective phylogenetic analysis and functions overlap between the two proteins [21]. Like Vpr, Vpx is packaged into lentiviral virions consistent with its involvement in early events of the lentiviral lifecycle counteracting host innate defences [22]. Like Vpr, Vpx interacts with host interactor protein damage-specific DNA binding protein 1 (DDB1)-Cullin4A (CUL4A)-associated factor 1 (DCAF1) which promotes ubiquitination and drives recruitment of proteasome machinery to degrade target host proteins, most notably sterile alpha motif and histidine-aspartate domain containing protein 1 (SAMHD1) and the human silencing hub (HUSH) complex, in order to enhance virus replication [23–26].

Here we demonstrate that Vpx is a broad inhibitor of NF-κB activation and pro-inflammatory gene expression active against diverse NF-κB agonists, including during virus infection. Vpx recruits p65 and inhibits nuclear translocation independently of Vpx-cofactor DCAF1. We found that this novel DCAF1-independent phenotype is conserved for all Vpx-encoding lentiviruses tested. We propose that Vpx has evolved to suppress inflammatory signals from a broad range of inflammatory and/or defensive stimuli which would otherwise limit transmission and ongoing replication of Vpx bearing viruses [27].

## Results

### Vpx is a broad antagonist of NF-κB activation

**Fig. 1:**
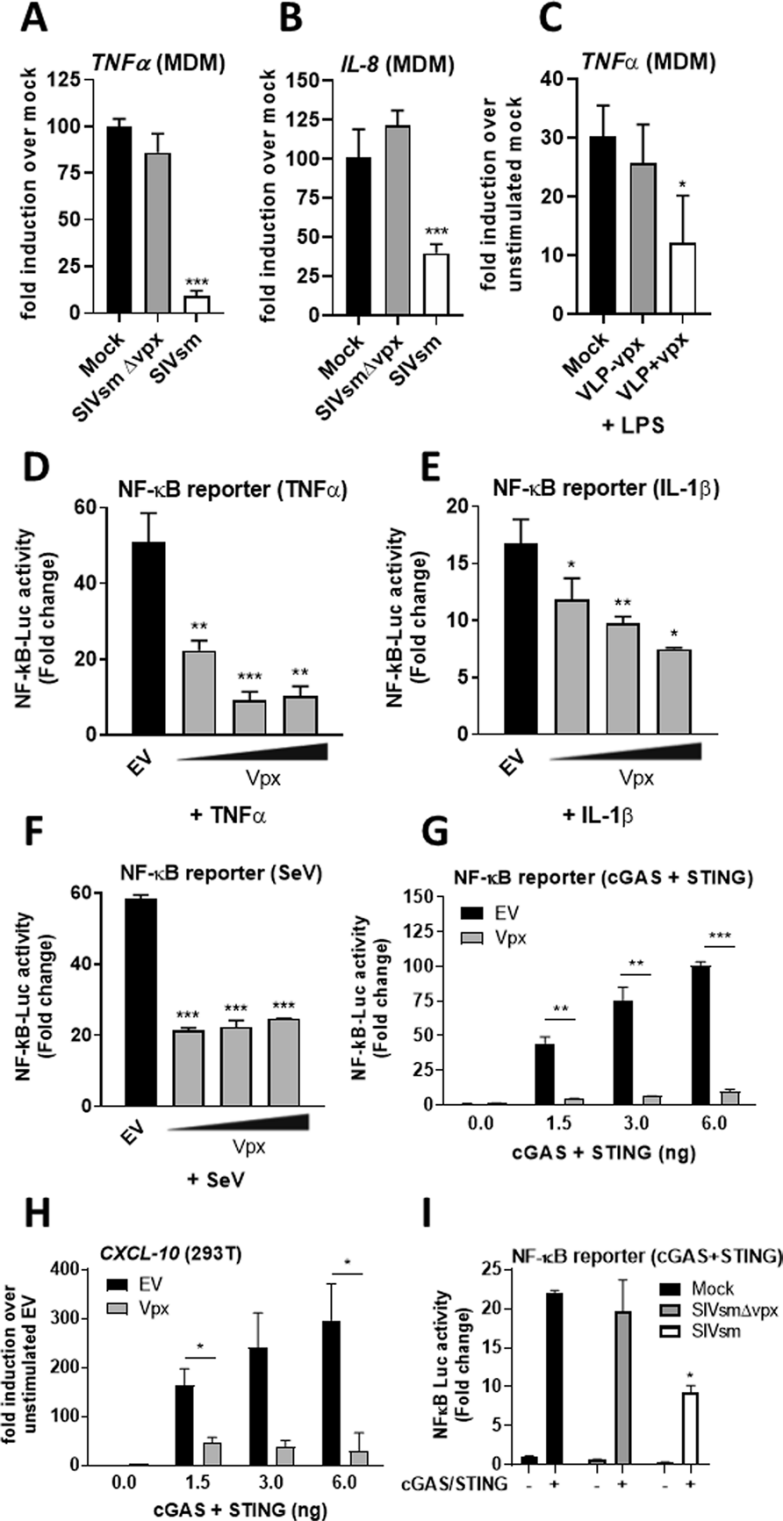
Vpx is a broad antagonist of NF-κB. **A** *TNF-α* qRT‐PCR from primary human monocyte-derived macrophages (MDM) infected for 48 h with SIVsm or SIVsmΔVpx (1.5 U/ml RT). **B** *IL-8* qRT‐PCR from MDM infected for 48 h with SIVsm or SIVsmΔVpx (1.5 U/ml RT). **C** *TNF-α* qRT‐PCR from MDM transduced for 24 h with VLPs −/+ Vpx (3 U/ml RT) followed by stimulation with 1 ng/ml LPS for 24 h. **D** NF-κB reporter activity from HEK293T cells transfected for 24 h with 25, 50 or 100 ng SIVmac Vpx or EV control (100 ng) per well and then stimulated for 8 h with 10 ng/ml TNF-α. **E** NF-κB reporter activity from HEK293T cells transfected for 24 h with 25, 50 or 100 ng SIVmac Vpx or EV control (100 ng) per well and then stimulated for 8 h with 1 ng/ml IL-1β. **F** NF-κB reporter activity from HEK293T cells transfected for 24 h with 25, 50 or 100 ng SIVmac Vpx or EV control (100 ng) per well and then stimulated for 12 h with 2.0 HA U/ml Sendai virus (SeV). **G** NF-κB reporter activity from HEK293T cells co-transfected for 24 h with 50 ng SIVmac Vpx or EV control plus 0, 1.5, 3 or 6 ng each of FLAG-cGAS and FLAG-STING per well. **H** *CXCL-10* qRT-PCR from HEK293T cells co-transfected for 24 h with 50 ng SIVmac Vpx or EV control plus 0, 1.5, 3 or 6 ng each of FLAG-cGAS and FLAG-STING per well. **I** NF-κB reporter activity from HEK293T cells transfected for 24 h with 1.5 ng FLAG-cGAS and FLAG-STING per well and then infected for 24 h with SIVsm or SIVsmΔVpx (1.0 U/ml RT). Data are mean ± SD, *n* = 3, representative of at least 3 repeats. Statistical analyses were performed using Student’s *t*‐test, with Welch’s correction where appropriate. *P < 0.05, **P < 0.01, ***P < 0.001

Whilst investigating innate immune responses to SIVsm lineage viruses in primary human immune cells we noted that although wild-type SIVsm infection of human monocyte-derived macrophages (MDM) did not induce NF-κB-dependent gene expression at the doses tested, basal expression of NF-κB-dependent genes such as tumour necrosis factor (TNF)α (Fig. 1A) and IL-8 (Fig. 1B) were significantly reduced in SIVsm-infected, compared to mock-infected, cells. This inhibition was not observed during infection with a virus lacking Vpx (SIVsmΔvpx) (Fig. 1A, B), despite similar infection levels (Additional file 1: Fig. S1A). We also found that Vpx delivered by genome-free virus-like particles (VLPs) antagonised NF-κB-dependent gene expression activated by lipopolysaccharide (LPS) treatment of MDM (Fig. 1C), indicating that Vpx could antagonise NF-κB activation driven by exogenous non-viral agonists. To study this inhibitory activity further we turned to reporter gene assays in HEK293T cells transiently expressing Vpx and an NF-κB-sensitive luciferase construct. Using this system we found that Vpx antagonised NF-κB activation in response to a broad range of stimuli including cytokines TNFα (Fig. 1D) and interleukin 1β (IL-1β, Fig. 1E), activation of RNA sensing pathways by Sendai virus (SeV, Fig. 1F) and activation of DNA sensing by transient over-expression of cGAS and STING (Fig. 1G). Inhibition of NF-κB downstream of cGAS/STING was further confirmed by measuring transcripts for NF-κB-dependent gene *CXCL-10* by qRT-PCR (Fig. 1H). Antagonism of DNA sensing-induced NF-κB activation by Vpx was dose-dependent (Additional file 1: Fig S1B) and was not due to inhibition of cGAS or STING expression (Additional file 1: Fig S1C, D) or cGAS/STING degradation (Additional file 1: Fig. S1B). In contrast to the effect of Vpx on NF-κB-dependent gene expression, transient co-expression of Vpx with cGAS/STING slightly increased activation of an IRF-3-driven luciferase reporter bearing the *IFIT-1* promoter (also known as *ISG56*, Additional file 1: Fig S1E, F), as well as *IFIT-1* mRNA expression (Additional file 1: Fig S1G). Finally, we demonstrated that infection of HEK293T cells with SIVsm inhibited cGAS/STING-dependent NF-κB reporter activation, whilst infection with SIVsmΔvpx did not (Fig. 1I), despite equivalent levels of infection (Additional file 1: Fig S1H). Together these data demonstrated Vpx to be a broad antagonist of NF-κB activated by both cognate virus infection and by exogenous stimuli. Further, in the case of DNA sensing, Vpx specifically inhibited NF-κB activation without affecting the activation of IRF3, demonstrating the specificity of this activity.

### Vpx inhibits NF-κB at the level, or downstream, of p65

**Fig. 2:**
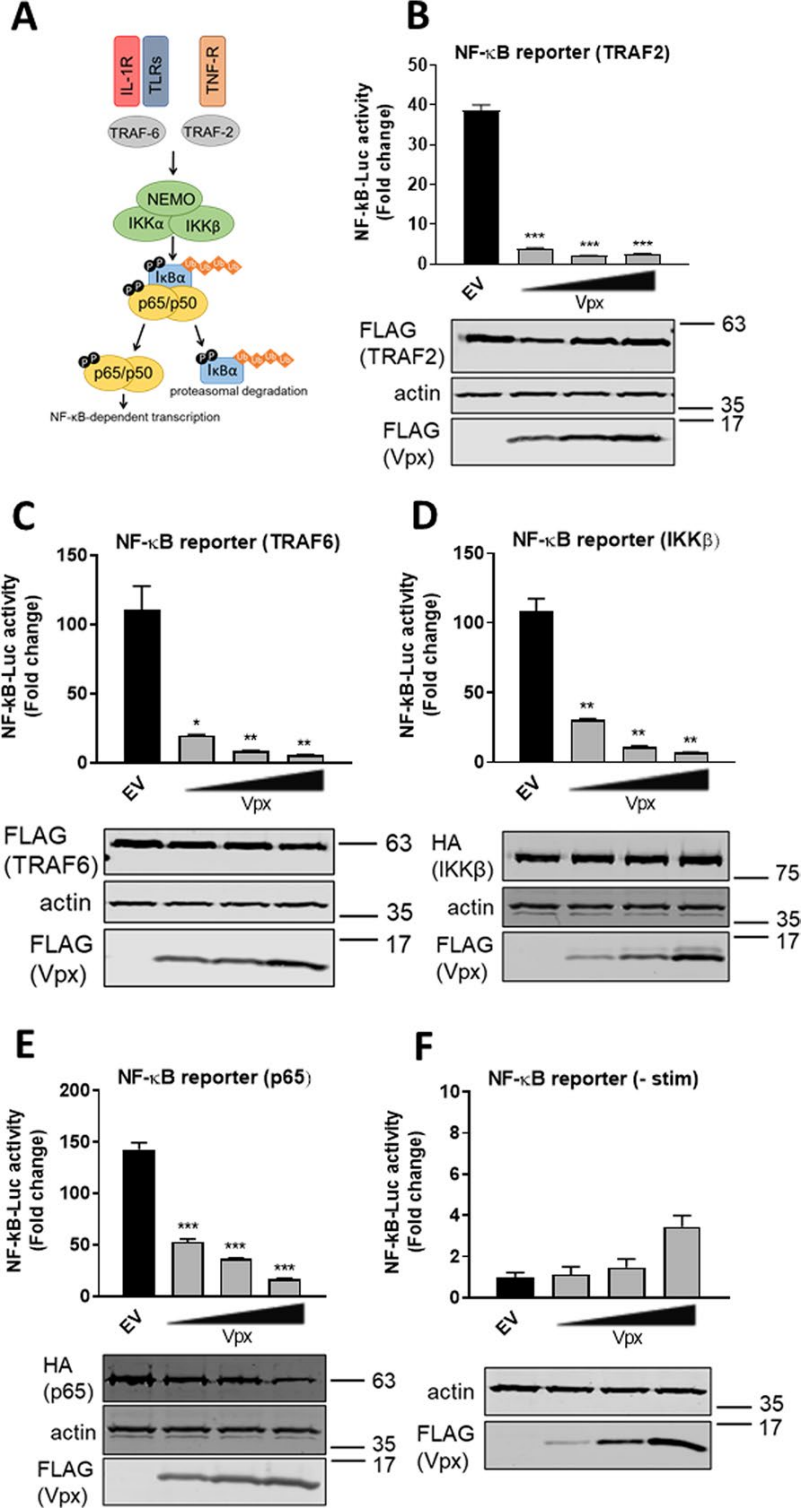
Vpx inhibits NF-κB at the level, or downstream, of p65. **A** Schematic of NF-κB activation downstream of the TNF receptor (TNFR), IL-1 receptor (IL-1R) and Toll-like receptors (TLRs). **B** NF-κB reporter activity and representative immunoblot from HEK293T cells co-transfected for 24 h with 25–100 ng SIVmac Vpx or EV control (100 ng) and 25 ng of TRAF2. Vpx and TRAF2 expression was detected with an anti-FLAG antibody. **C** NF-κB reporter activity and representative immunoblot from HEK293T cells co-transfected for 24 h with 25–100 ng SIVmac Vpx or EV control (100 ng) and 25 ng of TRAF6. Vpx and TRAF6 expression was detected with an anti-FLAG antibody. **D** NF-κB reporter activity and representative immunoblot from HEK293T cells co-transfected for 24 h with 25–100 ng SIVmac Vpx or EV control (100 ng) and 50 ng of IKKβ. IKKβ and Vpx expression were detected with anti-HA and anti-FLAG antibodies respectively. **E** NF-κB reporter activity and representative immunoblot from HEK293T cells co-transfected for 24 h with 25–100 ng SIVmac Vpx or EV control (100 ng) and 25 ng of p65. p65 and Vpx expression were detected with anti-HA and anti-FLAG antibodies respectively. **F** NF-κB reporter activity and representative immunoblot from HEK293T cells co-transfected for 24 h with 25–100 ng SIVmac Vpx or EV control (100 ng) in the absence of stimulation. Vpx expression was detected with an anti-FLAG antibody.Data are mean ± SD, *n* = 3, representative of at least 4 repeats. Statistical analyses were performed using Student’s *t*‐test, with Welch's correction where appropriate. *P < 0.05, **P < 0.01, ***P < 0.001

To gain mechanistic insight into Vpx antagonism of NF-κB, we undertook pathway mapping in HEK293T cells transiently expressing an NF-κB-sensitive luciferase reporter (Fig. 2A). Consistent with the ability of Vpx to inhibit NF-κB activation downstream of diverse stimuli, Vpx inhibited NF-κB activation by expression of adaptor proteins TNF⍺-activated tumour necrosis factor receptor-associated factor 2 (TRAF2; Fig. 2B), and IL-1β and Toll-like receptor (TLR)-activated TRAF6 (Fig. 2C), the kinase inhibitor of κB (IκB) kinase β (IKKβ; Fig. 2D) and also downstream of exogenous p65 expression (Fig. 2E). Overexpression of Vpx alone in the absence of co-stimulation had little effect on NF-κB reporter activity, except at the highest dose where reporter activity was slightly activated (Fig. 2F). Furthermore, Vpx did not significantly alter the expression of the co-transfected activating proteins, as assessed by immunoblotting (Fig. 2B–E). Together these data suggested antagonism of NF-κB signalling at or after p65 subunit activation.

### Vpx inhibition is independent of DCAF1, SAMHD1 and HUSH

**Fig. 3:**
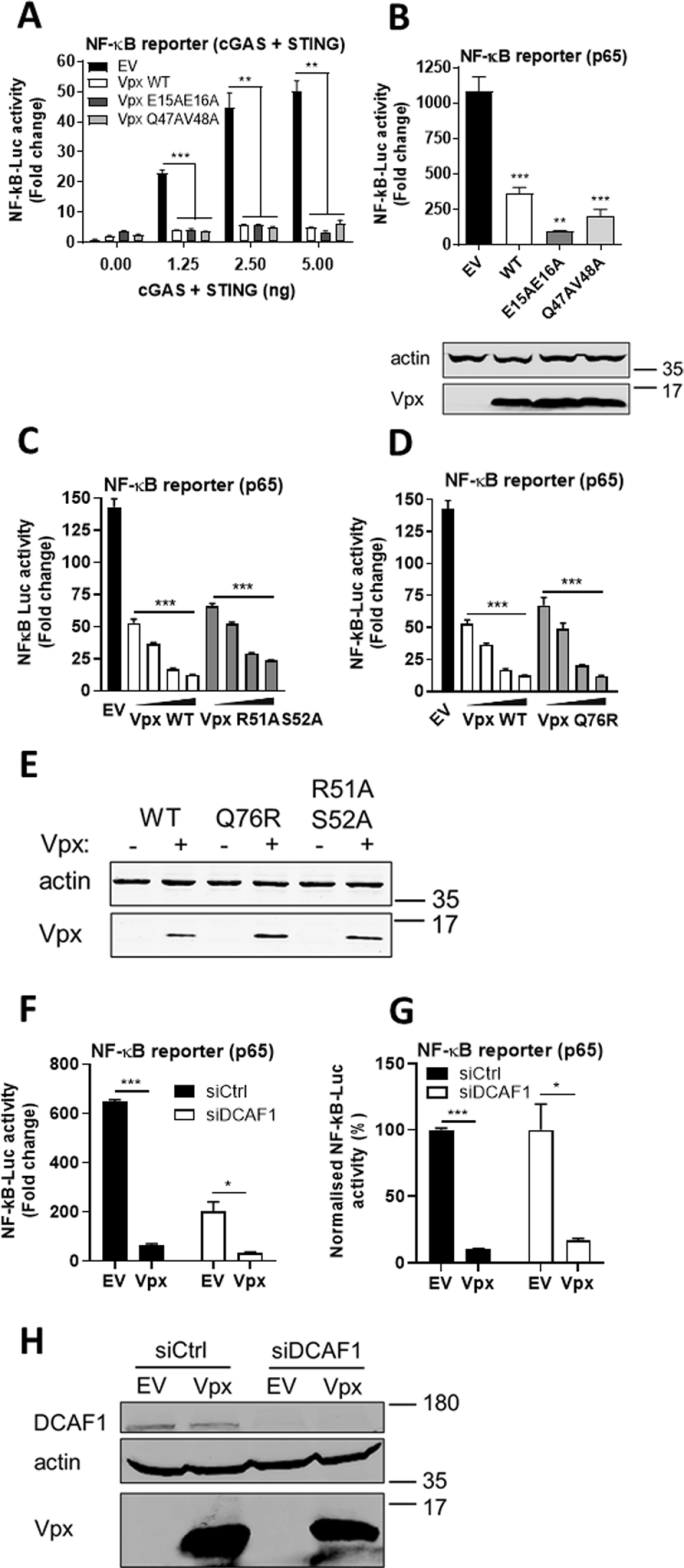
Vpx-mediated inhibition of NF-κB is independent of DCAF1, SAMHD1 and HUSH. **A** NF-κB reporter activity from HEK293T cells co-transfected for 24 h with 50 ng SIVmac WT, E15AE16A SAMHD1 mutant or Q47AV48A HUSH mutant Vpx or EV control plus 0, 1.25, 2.5 or 5 ng each of FLAG-cGAS and FLAG-STING per well. **B** NF-κB reporter activity from HEK293T cells co-transfected for 24 h with 50 ng SIVmac WT, E15AE16A SAMHD1 mutant or Q47AV48A HUSH mutant Vpx or EV control plus 25 ng p65 per well. Immunoblot detecting Vpx mutants using an antibody against the FLAG tag and actin. **C** NF-κB reporter activity from HEK293T cells co-transfected for 24 h with 12.5, 25, 50 or 100 ng SIVmac WT or R51AS52A STING mutant Vpx or EV control plus 25 ng p65 per well. **D** NF-κB reporter activity from HEK293T cells co-transfected for 24 h with 12.5, 25, 50 or 100 ng SIVmac WT or Q76R DCAF1 mutant Vpx or EV control plus 25 ng p65 per well. **E** Immunoblot from NF-κB reporter activity in HEK293T cells from **C**, **D** detecting Vpx mutants using an antibody against the FLAG tag and actin. The highest transfection dose was selected. **F** NF-κB reporter activity from HEK293T cells previously transfected for 48 h with siRNA against DCAF1 (siDCAF1) or Ctrl siRNA (siCtrl) and then co-transfected for 24 h with 50 ng SIVmac or EV control plus 25 ng p65 per well. **G** Normalised NF-κB reporter activity from **F**. **H** Immunoblot for DCAF1 depletion in NF-κB reporter activity in HEK293T cells from **F**, **G** detecting DCAF1, actin and Vpx using a Vpx antibody. Data are mean ± SD, *n* = 3, representative of at least 3 repeats. Statistical analyses were performed using Student’s *t*‐test, with Welch’s correction where appropriate. *P < 0.05, **P < 0.01, ***P < 0.001

Vpx interacts with a variety of host cellular proteins [28]. We used mutagenesis and depletion experiments to determine if these binding partners contributed to NF-κB inhibition. Vpx mutants deficient for antagonism of SAMHD1 (E15A E16A) and HUSH complex (Q47A V48A) retained capacity to antagonise gene expression activated by cGAS/STING (Fig. 3A) or p65 expression (Fig. 3B) [24, 29]. A mutant of Vpx that was recently described to prevent interaction with STING (R51AS52A) also still antagonised gene expression activated by p65 expression in a dose-dependent manner as effectively as wild-type Vpx (Fig. 3C). This was also true for Vpx Q76R, which is deficient for DCAF1 binding, suggesting DCAF1 independence for this activity (Fig. 3D) [23, 29, 30]. All mutant Vpx proteins were expressed at similar levels to wild-type (Fig. 3E). Concordantly, Vpx inhibited NF-κB activation in HEK293T cells depleted of DCAF1 by siRNA (Fig. 3F, G). Interestingly depletion of DCAF1 itself consistently reduced NF-κB reporter activity downstream of p65 over-expression in the empty vector (EV) control, but inhibition of the remaining signal was still observed by Vpx and this was to the same degree as in siCtrl cells (see normalised data in Fig. 3F). Depletion of DCAF1 protein to undetectable levels was confirmed by immunoblotting (Fig. 3H). These data suggest that Vpx antagonism of NF-κB signalling is independent of DCAF1 and associated Vpx interactome.

### Vpx interacts with p65

**Fig. 4:**
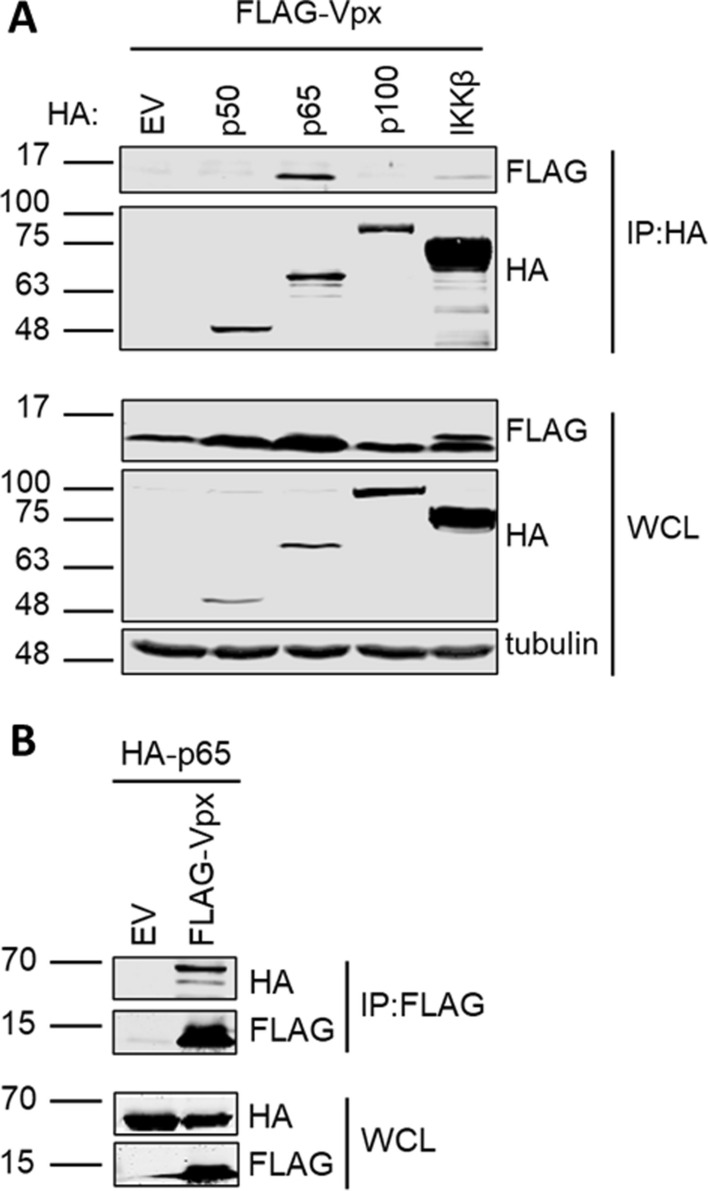
Vpx interacts with p65. **A** Immunoblot from co-immunoprecipitation assay performed from HEK293T cells co-transfected with FLAG-SIVmac Vpx and HA-tagged p50, p65, p100, IKKβ or an EV control. Whole cell lysates (WCL) were probed with FLAG, HA and tubulin antibodies and immunoprecipitates (IP) were probed for FLAG and HA following immunoprecipitation with anti-HA beads. **B** Immunoblot from co-immunoprecipitation assay performed from HEK293T cells co-transfected with HA-p65 and FLAG-SIVmac Vpx or an EV control. WCL and IP samples that had been incubated with anti-FLAG beads were probed with HA and FLAG antibodies. Data are from a representative experiment repeated 4 times

Given that Vpx could inhibit NF-κB induced gene expression by p65 over-expression we tested whether Vpx might bind NF-κB p65 directly. We co-expressed HA-tagged NF-κB proteins from both class I (p100 and p50) and II (p65) as well as IKKβ with FLAG-tagged Vpx in HEK293T cells. Indeed, immunoprecipitation of HA-tagged p65 specifically co-immunoprecipitated Vpx (Fig. 4A). This result was confirmed by reciprocal co-immunoprecipitation of HA-p65 on immunoprecipitation of FLAG-Vpx with anti-FLAG antibody (Fig. 4B).

### Vpx blocks p65 nuclear translocation

**Fig. 5:**
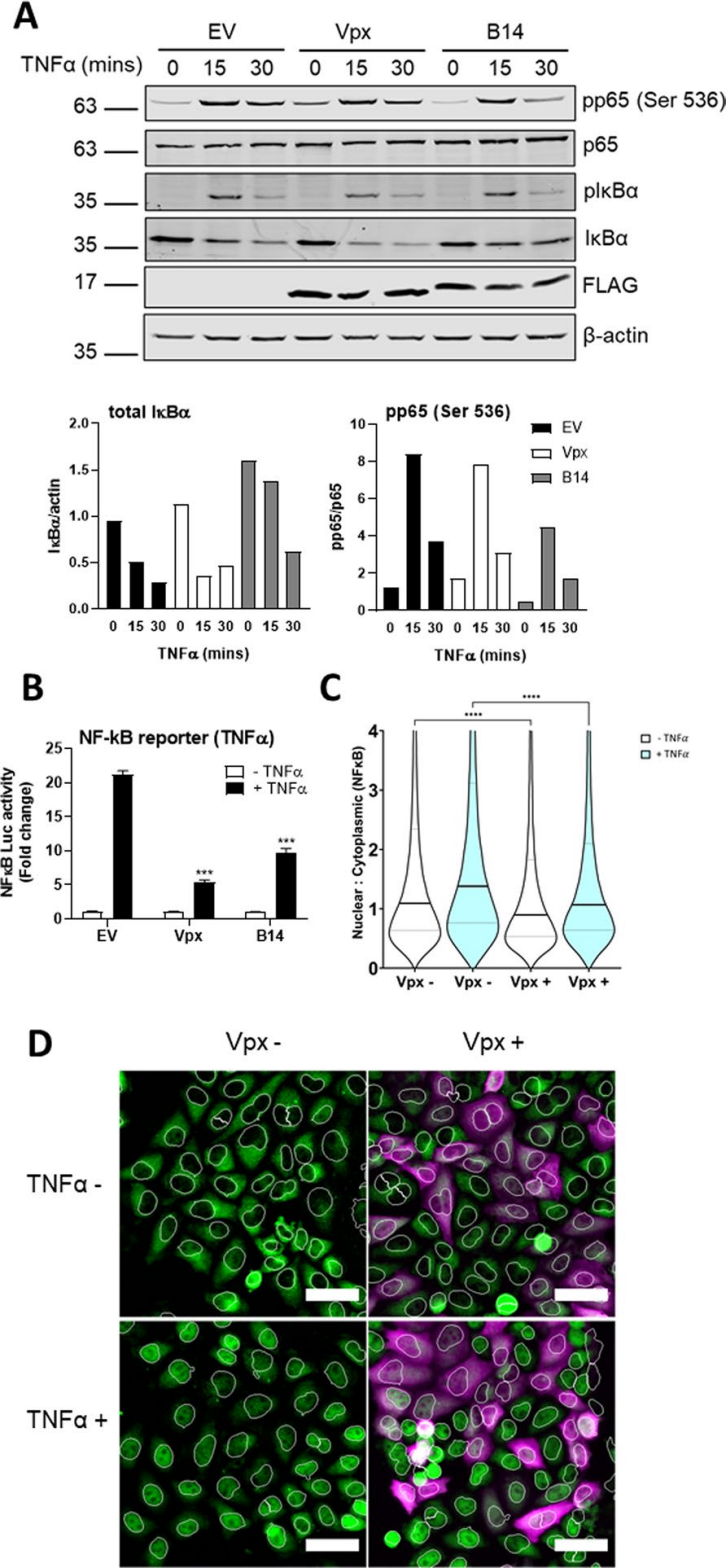
Vpx blocks p65 nuclear translocation. **A** Immunoblot from HEK293T cells that had been transfected for 24 h with 2 µg FLAG-tagged SIVmac Vpx, vaccinia virus protein B14 or EV control and stimulated for 0, 15 or 30 min with 50 ng/ml TNFα. Blots were probed with antibodies against total p65, phosphorylation of p65 on serine 536 (pp65), total IκBα, phosphorylated IκBα (pIκBα), FLAG for Vpx and B14 expression and actin. Signal intensity for total IκBα normalised to actin and pp65 normalised to total p65 are shown underneath. **B** NF-κB reporter activity from HEK293T cells transfected in parallel with the experiment from **A** and stimulated with 50 ng/ml TNFα for 8 h. Statistical analyses were performed using Student’s *t*‐test, with Welch’s correction where appropriate. ***P < 0.001. **C** Single cell analysis quantifying the Integrated Nuclear Intensity of NF-κB p65 in HeLa cells transfected with FLAG-tagged SIVmac Vpx or EV control and stimulated for 0 and 30 min with 50 ng/ml TNFα. Horizontal lines indicate the mean. Kruskall–Wallis test with Dunn’s multiple comparison, ****P < 0.0001. **D** Representative example of immunofluorescence staining of NF-κB p65 (green) after FLAG-tagged SIVmac Vpx or EV control transfection and stimulated for 0 and 30 min with 50 ng/ml TNFα. FLAG-tagged Vpx (magenta). Nuclei are outlined in white. Data in 5A are from a representative experiment repeated 3 times. Data in 5B are mean ± SD, *n* = 3, representative of 3 repeats

To further characterise the impact of Vpx on p65 at the endogenous level we performed assays measuring p65 phosphorylation and nuclear translocation. Phosphorylation of p65 at serine 536 was readily observed in HEK293T cells stimulated with TNF⍺ at 15 and 30 min post-stimulation in Vpx-transfected cells, indicating that interaction of Vpx with p65 did not interfere with this stimulation-induced phosphorylation event (Fig. 5A). Total p65 levels were also unaffected by Vpx expression (Fig. 5A), further supporting a model in which inhibition of p65 by Vpx is independent of DCAF1 and concordantly non-degradative (Fig. 3D–F). Furthermore, other hallmarks of NF-κB activation such as IκBα phosphorylation and degradation were also unaffected by Vpx expression (Fig. 5A). Conversely, as a control, vaccinia virus protein B14, an inhibitor of the IKK complex [31], prevented degradation of IκB⍺ and also reduced phosphorylation of p65, particularly at 30 min post-TNF⍺ treatment (Fig. 5A). NF-κB inhibition by Vpx and B14 was demonstrated in a parallel reporter gene assay performed using the same conditions as the phospho-blot assay (Fig. 5B). Despite phosphorylation of p65 at serine 536 in the presence of Vpx, nuclear translocation of this transcription factor was blocked by Vpx in TNF⍺-treated cells (Fig. 5C, D) explaining inhibition of NF-κB-dependent transcription by inhibition of NF-κB nuclear transport.

### Inhibition of NF-κB is conserved amongst Vpx species variants

**Fig. 6:**
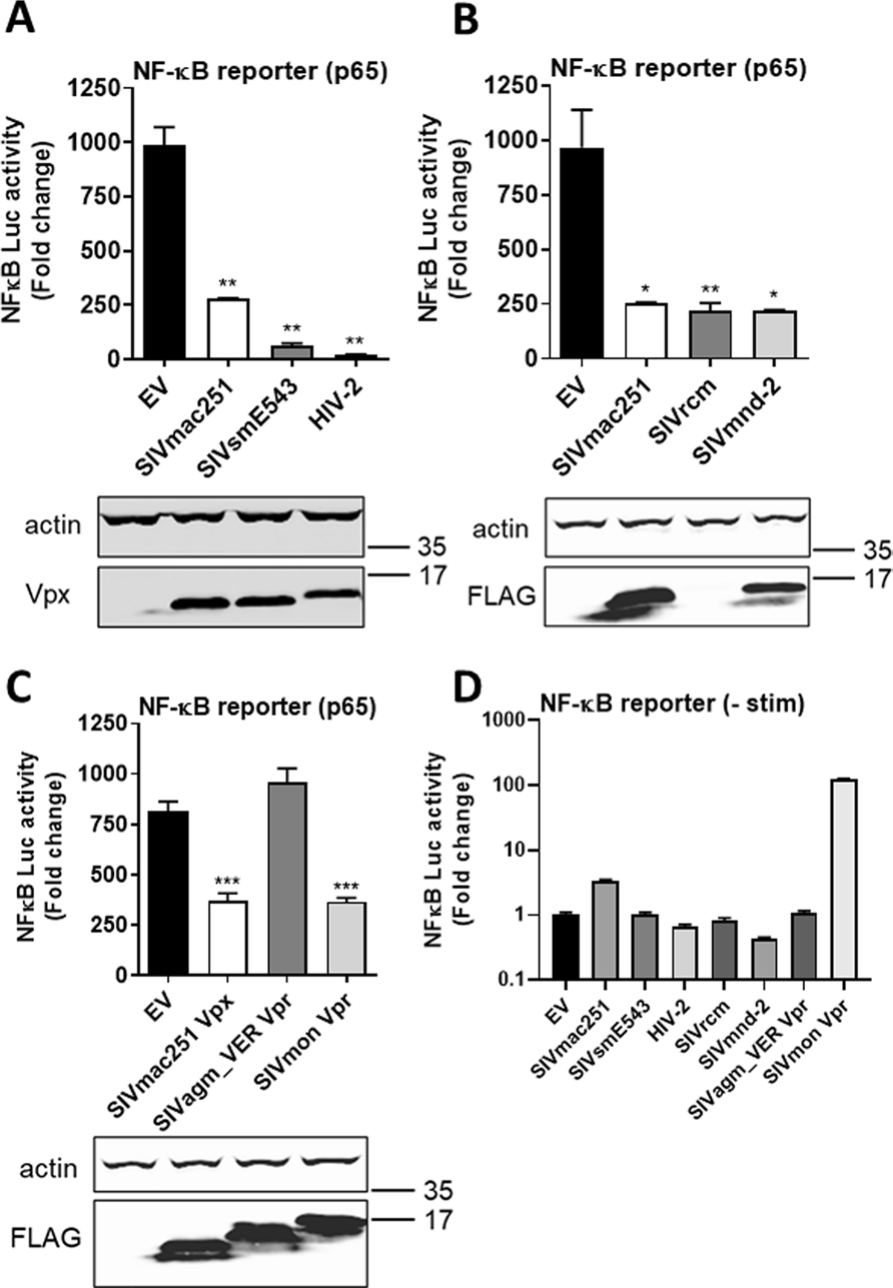
Inhibition of NF-κB is conserved amongst Vpx species variants. **A** NF-κB reporter activity and immunoblot from HEK293T cells co-transfected for 24 h with 50 ng Vpx proteins from SIVmac strain 251, SIVsm strain E543 or HIV-2 or EV control and 50 ng p65 per well. Vpx expression was detected using an anti-Vpx antibody. **B** NF-κB reporter activity and immunoblot from HEK293T cells co-transfected for 24 h with 50 ng FLAG-tagged Vpx proteins from SIVmac strain 251, SIVrcm or SIVmnd-2 or EV control and 50 ng p65 per well. Vpx expression was detected using an anti-FLAG antibody. **C** NF-κB reporter activity and immunoblot from HEK293T cells co-transfected for 24 h with 50 ng FLAG-tagged Vpx protein from SIVmac strain 251 or FLAG-tagged Vpr proteins from SIVagm_VER or SIVmon or EV control and 50 ng p65 per well. Vpx/Vpr expression was detected using an anti-FLAG antibody. **D** NF-κB reporter activity from HEK293T cells co-transfected for 24 h with 50 ng FLAG-tagged Vpx or Vpr proteins in the absence of stimulation. Data are mean ± SD, *n* = 3, representative of at least 3 repeats. Statistical analyses were performed using Student’s *t*‐test, with Welch’s correction where appropriate. *P < 0.05, **P < 0.01, ***P < 0.001

Vpr/Vpx proteins capable of degrading SAMHD1 are encoded by diverse primate lentiviruses including HIV-2 (Additional file 1: Fig. S2A) [32]. To determine whether inhibition of NF-κB was a conserved Vpx feature we cloned a series of diverse Vpx variants and tested their ability to suppress p65-driven NF-κB reporter gene activation in HEK293T cells. In agreement with data obtained using SIVsm infection (Fig. 1A, B, I), expression of Vpx from SIVsm strain E543 and HIV-2 inhibited NF-κB reporter activity similarly to Vpx from SIVmac (Fig. 6A). The other major clade of lentiviruses encoding genes commonly referred to as Vpx, besides the SIVsm/HIV-2 lineage, are derived from Red Capped Mangabeys and Mandrills (Additional file 1: Fig. S2A) and Vpx from these species also demonstrated anti-NF-κB reporter activity at or below the level of p65 activation (Fig. 6B). SIVrcm Vpx expression was significantly reduced compared to other Vpx species, as has been previously documented, requiring immunoblotting from a more concentrated lysate to demonstrate expression (Additional file 1: Fig. S2B) [33]. Finally we tested the Vpr proteins with SAMHD1-degrading activity from lentiviruses infecting vervet (SIVagm_VER) and mona monkeys (SIVmon) and found that whilst SIVagm_VER Vpr did not inhibit NF-κB downstream of p65, SIVmon Vpr did (Fig. 6C). Both Vpr proteins expressed well in this assay. Overexpression of the various Vpx/Vpr proteins did not alter NF-κB reporter activity in the absence of stimulation (Fig. 6D), except for a small activation with SIVmac Vpx and a very strong activation of the NF-κB reporter by Vpr from SIVmon, as has previously been observed [34]. Overall this suggests that inhibition of NF-κB is a conserved feature of Vpx, mapping at the level, or downstream of p65 activation.

## Discussion

In this study we evidence Vpx as an inhibitor of NF-κB signaling activated by diverse agonists (Fig. 1). Inhibition mapped to NF-κB family member p65 (Fig. 2) and was unrelated to Vpx interaction with known cellular partners including SAMHD1, HUSH complex, STING or DCAF1 (Fig. 3). Vpx co-immunoprecipitated with p65, but not NF-κB proteins p50 or p100 (Fig. 4). Vpx did not prevent p65 phosphorylation, a marker of activation, but rather inhibited p65 nuclear translocation (Fig. 5), explaining its broad activity against different NF-κB activating agonists. Consistent with independence from DCAF1, which recruits the CRL4 E3 ubiquitin ligase complex to drive target protein degradation, p65 was not degraded by Vpx (Fig. 5). Inhibition of p65 was found to be conserved amongst Vpx proteins from distantly related SIV, as well as HIV-2, and Vpr from SIVmon which, like Vpx, exhibits SAMHD1-degrading activity (Fig. 6).

These findings extend recent observations that Vpx binds STING to suppress NF-κB activation downstream of DNA sensing [30]. This preceding study did not explore the role of Vpx as an NF-κB signalling antagonist in the setting of cognate virus infection in the absence of pharmacological STING activation or test Vpx NF-κB antagonism against the full range of NF-κB agonists [30]. However, similar to this work, we also found that Vpx did not inhibit cGAS/STING-induced IRF3 activation (Additional file 1: Fig. S1E–G). However, we found that the proposed STING-binding Vpx mutant R51A S52A remained competent for NF-κB antagonism downstream of p65 activation. We suggest that in addition to manipulation of STING, Vpx directly targets p65 to prevent its nuclear translocation. Our findings are consistent with a recent study which identified Vpx-p65 interaction using unbiased mass spectrometry and further demonstrated inhibition of an NF-κB reporter by Vpx after p65 overexpression [35]. In contrast to our study, however, Landsberg and colleagues found that a mutant of Vpx that is deficient in DCAF1 binding (Vpx Q76A) had reduced ability to block NF-κB activity. In our study we have demonstrated that a different Vpx mutant (Q76R) that is defective for DCAF1 binding has similar NF-κB inhibitory activity (Fig. 3D) and expression levels (Fig. 3E) to WT Vpx and furthermore, depletion of DCAF1 with siRNA does not diminish the ability of Vpx to block NF-κB reporter activity (Fig. 3F, G).

An important future goal will be to further characterise the structural details of the Vpx-p65 interaction. This will aid the design of viral mutants that separate the various Vpx functions to allow their mechanistic dissection and evaluation of their importance in replication assays in different cell types. Crystal structures of Vpx-p65 complexes should be a tractable goal. Interestingly, we observed a double band for Vpx in the context of co-expressed IKKβ (Figs. 2D and 4A). It is tempting to speculate that this second band represents a phosphorylated form of Vpx, but whether Vpx is a direct target of IKKβ, or this additional band is due to promiscuous kinase activity through overexpression, also warrants further investigation.

The relationship between lentiviruses and NF-κB is complex because this transcription factor family has both anti-viral activities, e.g. downstream of DNA sensing, and pro-viral activities, as a key transcription factor in lentiviral promoters. Importantly, viral accessory proteins also have other pro-viral activities, mediated by manipulation of a further complex set of host pathways including manipulation of epigenetic regulation of transcription Vpx-HUSH [24, 25], Vpr-SLF2 [36], and regulation of NF-κB and IRF3 nuclear transport [17]. Thus, how Vpx impacts viral replication is very dependent on the nature of the assay, and the cells used. For example, during spreading infection in Jurkat T cells, which lack active SAMHD1 and cGAS, WT SIVmac and ∆vpx viruses replicate similarly, unless STING is activated with agonist (RR-S2 CDA), and then Vpx enhances infection, consistent with our data [30, 37]. The situation is certainly complex and incompletely understood. For example, rather than having a negative effect, through inhibiting NF-κB and therefore lentiviral transcription, available studies support the opposite phenomenon: Vpx degradation of HUSH enhances spreading infection of SIVmac in CEMx174 cells and HIV-1 proviral transcription in Jurkat cells with Vpx delivered by VLP [24, 25]. Indeed, Vpx degradation of HUSH to enhance transcription may compensate for transcription inhibition through NF-κB antagonism. It has been suggested that HIV-1 Vpr may be sequestered by Gag in infected cells allowing Vpr-inhibited pathways to reactivate [38]. This may also be true for Vpx, which is also incorporated into particles through Gag recruitment [39].

Classically, Vpx degradation of SAMHD1 has allowed Vpx-bearing VLP to rescue HIV-1 from SAMHD1 mediated inhibition of DNA synthesis. However, restored HIV-1 DNA synthesis cannot rescue viral replication because DNA typically activates DNA sensing by cGAS leading to induction of an antiviral state [40, 41]. Thus, although single round infection of macrophages is improved by Vpx, carrying Vpx does not tend to rescue HIV-2 or SIVsm replication in DNA sensing competent cells such as macrophages, irrespective of its anti-SAMHD1 activity. In vitro, SIVsm does not support spreading infection of cognate macrophages [42]. In peripheral blood samples from HIV-2 infected individuals, no HIV-2 proviral DNA was detected in circulating monocytes [43]. HIV-2 does not appear able to establish spreading infection of primary DCs or MDM in vitro [44–46]. The lack of sustained replication in myeloid cells is not thought to be related to cell entry or co-receptor preferences, and isolates that do not replicate in myeloid cells replicate in CD4+ T cells [47]. SAMHD1 degradation was detected in DCs exposed to HIV-2 despite the failure of the virus to establish infection [46]. On the other hand, HIV-1, which does not encode Vpx, replicates well in macrophages, infecting cells that are in a permissive G1 like state in which SAMHD1 is switched off, in order to bypass inhibition of reverse transcription [48]. One possibility is that Vpx-bearing viruses use Vpx to enhance replication in T cells rather than macrophages, manipulating NF-κB subtly enough to suppress anti-viral activity while retaining pro-viral transcription [49]. Dissecting the role of the various Vpx functions, and their impact on T-cell and macrophage replication may best be approached by Vpx mutants that separate activity against SAMHD1, HUSH and NF-κB measuring their effect on viral gene expression, replication and innate immune sensing in these different target cells. Derivation of clean mutants may best be approached by structural biology approaches as discussed above.

Pro-inflammatory cytokine secretion is activated during HIV-1 transmission in vivo [50]. Similar data are not available for Vpx-encoding viruses, but the ability to antagonise diverse anti-pathogen signalling likely benefits transmission, particularly across mucosal surfaces where sentinel myeloid cells will limit infection if activated for example, through type 1 interferon secretion [51–53]. Vpx mediated inhibition of NF-κB may also benefit the virus by antagonising signalling downstream of pattern recognition receptors including TLR7 and 8: both of which detect HIV-1 RNA and activate NF-κB [54].

Importantly, our study suggests an additional function for Vpx in manipulating cell biology via NF-κB and this should be taken into account when using Vpx experimentally to enhance transduction of lentiviral vectors [55, 56] and in in vivo applications, for example in lentivector gene transduction [57] and vaccine delivery [58].

## Conclusion

In conclusion we have discovered a novel mechanism by which lentiviruses antagonise NF-κB activation using accessory protein Vpx. We have found Vpx to have broad anti-NF-κB activity in that it suppresses NF-κB activation by a diverse series of agonists including LPS, cytokines (TNFα and IL-1β), RNA sensing activated by Sendai virus infection and DNA sensing activated by STING/cGAS expression. It is not clear why Vpx has more potent activity against some agonists than others (Fig. 1), but we hypothesise that this reflects differences in the NF-κB activation mechanism between the upstream agonists. Species-specific differences in Vpx activity likely reflect the different degree to which the Vpx interacts with the human p65 protein, and therefore efficiency of nuclear translocation and subsequent NF-κB-dependent gene expression. NF-κB inhibition was found to be conserved amongst Vpx proteins from distantly related SIV, as well as HIV-2 and Vpr from SIVmon. These findings extend our knowledge of how lentiviruses manipulate universal regulators of immunity to avoid the anti-viral sequelae of pro-inflammatory gene expression stimulated by both viral and extra-viral agonists. Further structural studies of p65 targeting by Vpx may yield translational insights in the form of novel pan-NF-κB inhibitors. Finally, our findings are also relevant to the gene therapy field where virus-like particle associated Vpx is routinely used to enhance vector transduction through antagonism of SAMHD1, and perhaps also through manipulation of other pathways such as NF-κB.

## Materials and methods

### Plasmids

Codon-optimised Vpx cDNAs were synthesised by GeneArt (Regensburg, Germany) and cloned into a pcDNA3.1 plasmid with or without an N-terminal FLAG tag using BamHI and NotI (see Table 1 for accession numbers). Point mutations were introduced into plasmids using site directed mutagenesis with overlapping forward and reverse primers, each bearing the mutation (see Table 2). SDM PCRs were performed using Pfu Turbo DNA Polymerase (Agilent, Santa Clara, CA) followed by DpnI digest (NEB, Ipswich, MA) according to the manufacturer’s instructions (Agilent). Successful insertion of the desired mutations was confirmed by sequencing. The NF-κB reporter plasmid containing five copies of an NF-κB response element fused to the firefly luciferase gene and TK-renilla control plasmid were obtained from Promega (Madison, WI). The IFIT1 (ISG56) promoter fused with firefly luciferase reporter construct was obtained from A. Bowie, Trinity College Dublin. Plasmids expressing HA-tagged p50, p65, p100 and IKKβ and FLAG-tagged cGAS and STING were generated by amplifying the relevant cDNAs and cloning them into a version of pcDNA3.1 with an N-terminal HA or FLAG tag with NotI and XbaI. Plasmids expressing TRAF2 and TRAF6 were obtained from A. Bowie, Trinity College Dublin.

**Table 1:** Accession numbers

Isolate	Accession number
SIVmac251	M19499
SIVsmE543	U72748
SIVrcm	AF349680
SIVmnd-2	AF328295
SIVagm_VER	M29975
SIVmon	AY340701
HIV-2	M31113

**Table 2:** Primers

Target	Sequence
*GAPDH*	Fwd 5′‐GGGAAACTGTGGCGTGAT‐3′ Rev 5′‐GGAGGAGTGGGTGTCGCTGTT‐3′
*CXCL-10*	Fwd 5′‐TGGCATTCAAGGAGTACCTC‐3′ Rev 5′‐TTGTAGCAATGATCTCAACACG‐3′
*TNF-α*	Fwd 5′-AGCCTCTTCTCCTTCCTGATCGTG-3′ Rev 5′-GGCTGATTAGAGAGAGGTCCCTGG-3′
*IFIT-1*	Fwd 5′-CCTCCTTGGGTTCGTCTACA-3′ Rev 5′-GGCTGATATCTGGGTGCCTA-3′
*IL-8*	Fwd 5′-GAGAGTGATTGAGAGTGGACCAC-3′ Rev 5′-CACAACCCTCTGCACCCAGTTT-3′
Vpx E15A E16A	Fwd 5′-CAATAGCGGCGCCGCAACCATCGAAG-3′ Rev 5′-CCAGGAGGGATTCTCTCTC-3′
Vpx Q47A V48A	Fwd 5′-GCTGATCTTCGCCGCGTGGCAGAGAAGCTGGG-3′ Rev 5′-TCGCGAGGCAGATGGTTC-3′
Vpx R51A S52A	Fwd 5′-GCTAATTTTCCAGGTTTGGCAAGCGGCCTGGGAATACTGG-3′ Rev 5′-CCAGTATTCCCAGGCCGCTTGCCAAACCTGGAAAATTAGC-3′
Vpx Q76R	Fwd 5′-GTGCCTGATCAGGAAAGCCCTGTTC-3′ Rev 5′-AGGTATCTGTACTTGGTG-3′

### Cell lines and primary cell preparation

HEK293T and HeLa cells were grown in Dulbecco’s modified Eagle’s medium (DMEM; Gibco, Amarillo, TX) supplemented with 10% fetal calf serum (FCS; Gibco) and penicillin–streptomycin (50 μg/ml) (Gibco). Peripheral blood mononuclear cells (PBMCs) were prepared from HIV seronegative donors (after informed consent was obtained), by density-gradient centrifugation (Lymphoprep, Axis-Shield, Dundee, UK). Monocyte-derived macrophages (MDM) were prepared by adherence with washing of non-adherent cells after 2 h, with subsequent maintenance of adherent cells in RPMI 1640 medium supplemented with 10% human serum and M-CSF (10 ng/ml, Peprotech, London, UK) for 3 days and then differentiated for a further 4 days in RPMI 1640 medium supplemented with 10% fetal calf sera without M-CSF.

### Agonists

Lipopolysaccharide (LPS), tumour necrosis factor-alpha (TNF-α) and interleukin-1 β (IL-1β) were obtained from Peprotech. Sendai virus was obtained from Charles River Laboratories, Wilmington, MA.

### Transfection and small interfering RNA (siRNA) interference

For dual luciferase reporter gene assays in HEK293T cell, 1.5 × 10^5^ cells/ml were seeded in 24 well plates and transfected with 5 ng luciferase reporter (IFIT1 or NF-κB-sensitive), 2.5 ng thymidine kinase renilla luciferase reporter (Promega), 0.5–200 ng empty or Vpx expressing pcDNA3.1, 1.5–6 ng pcDNA3.1 FLAG-cGAS and STING using 0.75 μl FuGENE 6 (Promega) and 20 µl Opti-MEM (Gibco). All transfections were topped up with an empty vector plasmid to equalise total amounts of DNA. 48 h later cells were lysed in passive lysis buffer (Promega), and firefly and renilla luciferase activities were measured using a Glomax luminometer (Promega). Expression of the thymidine kinase renilla luciferase reporter was used as a control for transfection efficiency between wells. The fold induction of the reporter activity was calculated by normalising each result to the luciferase activity of the unstimulated cells transfected with empty pcDNA3.1 (EV). For siRNA experiments HEK293T cells (1.5 × 10^5^ cells/ml) were seeded in 6-well plates and transfected with 150 nM siRNA using 4 μl Lipofectamine 2000 (Invitrogen) and 184 μl OptiMEM. 48 h later 2 × 10^5^ cells/ml were seeded in 24-well plate to carry out the reporter gene assays. siRNA target sequences:

- Control AAUUCUCCGAACGUGUCACGUACGUGACACGUUCGGAGAAUU
- DCAF1 CGGAGUUGGAGGAGGACGAUUUCGUCCUCCUCCAACUCCGUU

### Virus production and infection

VLPs were produced by transfecting T150 flasks of HEK293T cells with 8 μg of vesicular stomatitis virus‐G glycoprotein (VSV-G) expressing plasmid pMDG (Genscript, Piscataway, NJ) pMDG, 32 µg SIV4+ [59] and with or without 1 µg of pcDNA3.1 Vpx expression plasmid using Fugene 6 transfection reagent (Promega) according to the manufacturer’s instructions. A chimeric virus derived from primary isolate SIVsm(E543) was used to investigate the function of Vpx in the context of the original SIVsm lineage [60]. The chimera was made by inserting the *gag*, *pol* and accessory genes (*vif*, *vpr*, *vpx*) of SIVsm(E543) into an SIVmac239-based vector where a large deletion in e*nv* limits the vector to single-round infection of human cells and *GFP* is expressed in place of *nef*. SIVsm(E543) WT and Δvpx were produced by transfecting 10 µg pMDG and 25 µg SIVsm construct. Supernatants were harvested 48 and 72 h post-transfection and filtered through a 0.45 μm filter. All lentivectors and VLPs were DNAse treated (2 h at 37 °C, DNaseI, Sigma, St Louis, MO) before they were concentrated by ultracentrifugation in a Sorvall Discovery (Hitachi) at 23000 rpm for 2 h at 4 °C under vacuum conditions through a 20% sucrose cushion. The pellet was then resuspended in RPMI + 10% FCS and stored at − 80 °C. Viral preparations were quantified by qPCR using a SYBR Green‐based product‐enhanced RT (SG‐PERT) assay as described to equilibrate viral dose [61]. MDM were infected in the presence of 8 µg/ml polybrene (Sigma) and for SIVsm, infection levels were determined 48 h later by enumerating GFP‐positive cells by flow cytometry using the FACS Calibur (BD, Franklin Lakes, NJ) and analysing with FlowJo software.

### Immunoblotting and immunoprecipitation

For immunoblotting cells were lysed in either passive lysis buffer (Promega) or cell lysis buffer (50 mM Tris pH 8, 150 mM NaCl, 1 mM EDTA, 10% (v/v) glycerol, 1% (v/v) Triton X100, 0.05% (v/v) NP-40 supplemented with protease inhibitors (Roche, Basel, Switzerland), and phosphatase inhibitors (Roche) for immunoblotting with phospho-specific antibodies. Lysates were clarified by centrifugation at 14,000×*g* for 10 min and boiled in 6× protein loading buffer, containing 50 mM Tris–HCl (pH 6.8), 2% (w/v) SDS, 10% (v/v) glycerol, 0.1% (w/v) bromophenol blue, 100 mM β-mercaptoethanol for 5 min. Proteins were separated by SDS-PAGE on 12% polyacrylamide gels and transferred to a Hybond ECL membrane (Amersham biosciences, Little Chalfont, UK) using a semi-dry transfer system (Biorad, Hercules, CA). Membranes were subsequently blocked by incubation for 1 h at room temperature in 5% (w/v) milk proteins + 0.01% (v/v) Tween-20 in PBS (PBST). The membranes were then incubated overnight at 4 °C with primary antibody (Ab) diluted in 5% (w/v) milk proteins in PBST. Primary antibodies were from the following sources: mouse anti‐β‐actin (Abcam, Cambridge, UK), mouse-anti-tubulin (EMD Millipore, Burlington, MA), mouse-anti-FLAG (Sigma), rabbit-anti-HA (Sigma), mouse-anti-p65 (Santa Cruz, Dalas, TX), rabbit-anti-phospho p65 (Ser 536) (Cell Signaling, Danvers, MA), rabbit-anti-IκBα (Cell Signaling), mouse-anti-phospho-IκBα (Cell Signaling), mouse-anti-Vpx raised against HIV-2 Vpx (NIH AIDS Reagents), rabbit-anti-DCAF1 (Bethyl, Montgomery, TX). Primary antibodies were detected with goat‐anti‐mouse/rabbit IRdye 800CW infrared dye secondary antibodies and membranes imaged using an Odyssey Infrared Imager (LI‐COR Biosciences, Lincoln, NE).For co-immunoprecipitation assays HEK293T cells were grown in 10 cm dishes and co-transfected with 5 µg of a plasmid expressing FLAG-tagged Vpx and 5 µg of a plasmid expressing HA-tagged p50, p65, p100, IKKβ or an empty vector (EV) control using polyethylenimine (Polysciences, Warrington, PA) according to the manufacturer’s instructions. After 24 h cells were lysed in lysis buffer (0.5 (v/v))% NP-40 in PBS supplemented with protease inhibitors (Roche) and phosphatase inhibitors (Roche), pre-cleared by centrifugation and incubated with 25 µl of mouse-anti-HA agarose beads (Millipore) or mouse-anti-FLAG M2 agarose affinity gel (Sigma) for 2–4 h. Immunoprecipitates were washed 3 times in 1 ml of lysis buffer and eluted from the beads by boiling in 20 µl of 2× protein loading buffer. Proteins were resolved by SDS-PAGE and detected by immunoblotting as described above.

### RNA extraction and quantitative real-time PCR (RT-PCR)

RNA was extracted using a total RNA purification kit (Norgen, Ontario, Canada) according to the manufacturer’s instructions. 1 µg RNA was used to synthesise cDNA using Superscript III reverse transcriptase (Invitrogen, Waltham, MA), also according to the manufacturer’s protocol. cDNA was diluted 1:5 in water and 2 μl was used for real‐time PCR using SYBR® Green PCR master mix (Applied Biosystems, Waltham, MA) and a Quant Studio 5 real‐time PCR machine (Applied Biosystems). Expression of each gene was normalised to an internal control (*GAPDH*), and these values were then normalised to mock/EV‐treated control cells to yield a fold induction.

### Nuclear translocation assay

#### Image acquisition

For nuclear translocation assays, HeLa cells (5 × 10^4^ cells/ml) were adhered in an Cellcarrier Ultra optical 96-well plate (PerkinElmer, Waltham, MA). Cells were washed three times with ice-cold PBS and fixed in 4% (v/v) paraformaldehyde. The cells were permeabilised in 0.1% (v/v) Triton X-100 in PBS, and blocked for 1 h in 10% (v/v) goat serum in PBS with 0.1% w/v BSA. The cells were stained with mouse-anti-p65 (Sigma) for 1 h followed by incubation with goat anti-mouse Alexa Fluor 488 secondary IgG antibody (Life Technologies, Carlsbad, CA). Cells were stained with anti-flag antibody for 1 h. Cells were incubated for 1 h with Phalloidin-568 in PBS, washed followed by incubation for 30 min with 1 µg/ml DAPI (4′,6-diamidino-2 phenylindole) was added per well to visualise DNA. Cells were washed with PBS three times between each step. Images were acquired using the WiScan® Hermes High-Content Imaging System (IDEA Bio-Medical, Rehovot, Israel) at magnification 10X/0.4NA. Four channel automated acquisition was carried out sequentially (DAPI/TRITC, GFP/Cy5). Images were acquired at 10X magnification, 100% density/80% well area resulting in 47 FOV/well.

#### Image analysis

NF-κB raw image channels were pre-processed using a batch rolling ball background correction in FIJI imagej software package prior to quantification [62]. Automated image analysis was carried out using the ‘Translocation’ module of the Athena Image analysis software (IDEA Bio-Medical, Rehovot, Israel). Firstly, nuclei were identified as primary objects by segmentation of the Hoechst33342 channel. Cells were identified as secondary objects by nucleus depended on segmentation of the Phalloidin-A488 channel. Cell cytoplasm was segmented by subtracting the nuclear objects mask from the cell masks. Vpx positive cells were identified by identifying Vpx IF signal (FLAG-A488)signal as independent granules. Vpx+ cells were determined by the presence of Vpx+ signal within a cell border applied to filter the segmented cell population. Intensity properties were calculated for the nuclei, cytoplasm and cell object populations. Nuclear:cytoplasmic ratio (NCR) was calculated as part of the pipeline by dividing the integrated intensity of the nuclei object by the integrated intensity of corresponding cytoplasm object. Data post-processing was carried out in Python programming languages using the Numpy and Pandas packages. An arbitrary cut-off of NCR of less than 0.01 and greater than 10 was applied to the data to filter outliers. 10,000 cells per condition, sampled in Python were plotted using Graphpad Prism 9.

### Phylogenetic analysis

*Vpr* and *vpx* sequences obtained from the Los Alamos database were manually aligned using the Seaview sequence editor [63]. Whilst *vpr* and *vpx* have undergone extensive insertions and deletions in various lineages, multiple alignment strategies consistently give the clades of interest strong support. Maximum-likelihood phylogenies were estimated using the General Time-Reversible model of nucleotide substitution with gamma-distributed rate variation across sites (GTR-gamma) implemented in RAxML 8 software [64]. Branch support was determined using 1000 bootstrap alignments. Phylogenies were visualized with the program FigTree (https://github.com/rambaut/figtree/releases). The vpr/vpx tree was midpoint-rooted. Branch lengths indicate the number of nucleotide substitutions per site.

### Statistical analysis

Statistical analyses were performed using an unpaired Student’s *t*‐test with Welch’s correction where variances were unequal. *P < 0.05, **P < 0.01, ***P < 0.001, ****P < 0.0001.

## Supplementary Information

**Additional file 1: Figure S1.** Vpx is a broad antagonist of NF-κB. **Figure S2.** Inhibition of NF-κB is conserved amongst Vpx species variants.

## Data Availability

All data generated or analysed during this study are included in this published article and its Additional files.

## Abbreviations

cGAS: Cyclic GMP-AMP synthase
DC: Dendritic cell
DCAF1: DDB1-Cullin4A-associated factor 1
DDB1: Damage-specific DNA binding protein 1
HIV: Human immunodeficiency virus
HUSH: Human silencing hub
IFN: Interferon
IL: Interleukin
IRF: Interferon regulatory factor
LPS: Lipopolysaccharide
MDM: Monocyte-derived macrophage
NCR: Nuclear:cytoplasmic ratio
NF-κB: Nuclear factor kappa B
PAMP: Pathogen-associated molecular pattern
PBMC: Peripheral blood mononuclear cells
PBST: Phosphate-buffered saline + 0.01% (v/v) Tween-20
RIG-I: Retinoic acid-inducible gene
SAMHD1: Sterile alpha motif and histidine-aspartate domain containing protein 1
SeV: Sendai virus
SG-PERT: SYBR green‐based product‐enhanced RT
SIV: Simian immunodeficiency virus
STING: Stimulator of interferon genes
TF: Transcription factor
TNF: Tumour necrosis factor
TRAF: Tumour necrosis factor receptor-associated factor 2
VLP: Virus-like particle
