# Supplementary material for: HIV-2/SIV Vpx antagonises NF-κB activation by targeting p65
Source: Retrovirology. 2022 Jan 24;19:2. doi: 10.1186/s12977-021-00586-w (PMC8785589; doi:10.1186/s12977-021-00586-w)
Supplement: Supplementary file 1 — Additional file 1: Figure S1. Vpx is a broad antagonist of NF-κB. Figure S2. Inhibition of NF-κB is conserved amongst Vpx species variants. [file 12977_2021_586_MOESM1_ESM.docx]

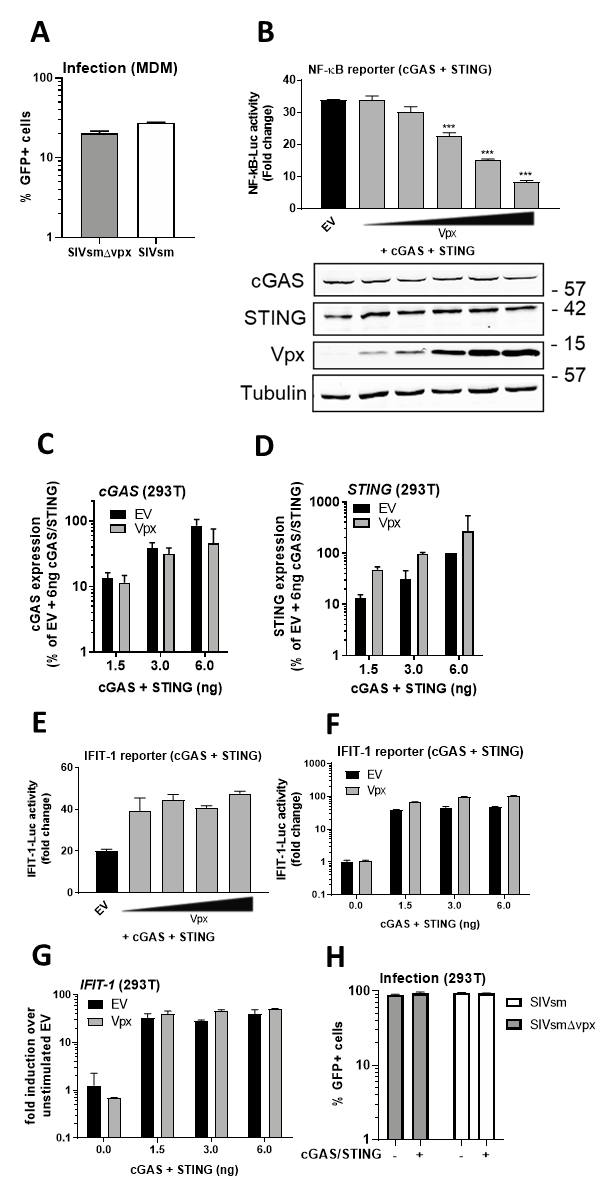


Figure S1. Vpx is a broad antagonist of NF-𝜅B

A: Infection data from Fig 1A and B. MDM infected for 48h with SIVsm or SIVsmΔVpx expressing GFP (1.5 U/ml RT).

B: NF-𝜅B reporter activity and immunoblot from HEK293T cells co-transfected for 24h with 1-100ng SIVmac Vpx or EV control (100ng) and 1.5ng each of FLAG-cGAS and STING per well. cGAS and STING were detected using a FLAG antibody.

C: *cGAS* qRT-PCR from HEK293T cells co-transfected for 24h with 50ng SIVmac Vpx or EV control plus 1.5, 3 or 6ng each of FLAG-cGAS and FLAG-STING per well.

D: *STING* qRT-PCR from HEK293T cells co-transfected for 24h with 50ng SIVmac Vpx or EV control plus 1.5, 3 or 6ng each of FLAG-cGAS and FLAG-STING per well.

E: IFIT-1 reporter activity from HEK293T cells co-transfected for 24h with 12.5-100ng SIVmac Vpx or EV control (100ng) and 1.5ng each of FLAG-cGAS and STING per well.

F: IFIT-1 reporter activity from HEK293T cells co-transfected for 24h with 50ng SIVmac Vpx or EV control plus 0, 1.5, 3 or 6ng each of FLAG-cGAS and FLAG-STING per well.

G: *IFIT-1* qRT-PCR from HEK293T cells co-transfected for 24h with 50ng SIVmac Vpx or EV control plus 0, 1.5, 3 or 6ng each of FLAG-cGAS and FLAG-STING per well.

H: Infection data from Fig 1I. HEK293T infected for 48h with SIVsm or SIVsmΔVpx expressing GFP (1.0 U/ml RT).

Data are mean ± SD, *n* = 3, representative of at least 3 repeats. Statistical analyses were performed using Student's *t*‐test, with Welch's correction where appropriate. ****P*< 0.001.


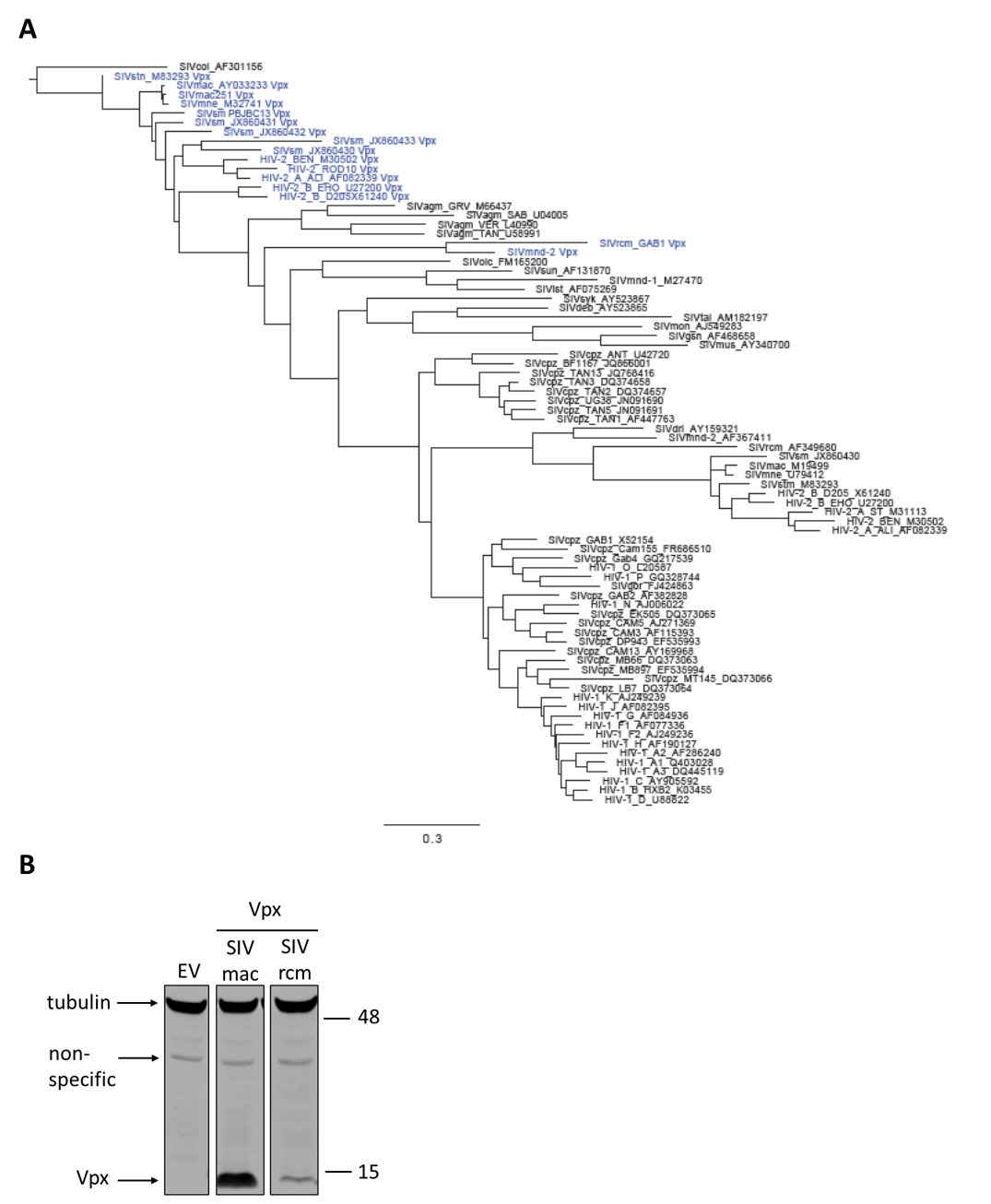


Figure S2. Inhibition of NF-𝜅B is conserved amongst Vpx species variants

A: Maximum likelihood phylogeny of primate lentiviruses *vpr* and *vpx* genes. Vpx sequences are coloured blue; vpr sequences are coloured black. Horizontal branch lengths are shown to scale. Scale bar represents 0.3 nt substitutions per site.

B: Immunoblot from HEK293T cells transfected for 24h with 1000ng EV, SIVmac Vpx or SIVrcm Vpx. Immunoblotting was performed from wells of a six well plate lysed in 100μl. Blots were probed with antibodies against FLAG for Vpx expression and tubulin. Samples shown are from the same gel with irrelevant samples cropped out.
